# The prevalence of bacteremia in out of hospital cardiac arrest patients presenting to the emergency department of a tertiary care hospital

**DOI:** 10.1080/07853890.2021.1953703

**Published:** 2021-07-20

**Authors:** Gilbert Abou Dagher, Ralph Bou Chebl, Rawan Safa, Mohammad Assaf, Nadim Kattouf, Karim Hajjar, Christopher El Khuri, Iskandar Berbari, Maha Makki, Mazen El Sayed

**Affiliations:** a Department of Emergency Medicine, American University of Beirut, Beirut, Lebanon; b Department of Internal Medicine, American University of Beirut, Beirut, Lebanon

**Keywords:** Outcome, mortality, infection, antibiotics, prophylaxis, cardiac arrest, bacteraemia, emergency medicine

## Abstract

**Background:**

Out-of-hospital cardiac arrest (OHCA) remains one of the most common causes of death. There is a scarcity of evidence concerning the prevalence of bacteraemia in cardiac arrest patients presenting to the Emergency Department (ED). We aimed to determine the prevalence of bacteraemia in OHCA patients presenting to the ED, as well as study the association between bacteraemia and in-hospital mortality in OHCA patients. In addition, the association between antibiotic use during resuscitation and in-hospital mortality was examined.

**Methods and results:**

This was a study of 200 adult OHCA patients who presented to the ED between 2015 and 2019. Bacteraemia was confirmed if at least one of the blood culture bottles grew a non-skin flora pathogen or if two blood culture bottles grew a skin flora pathogen from two different sites. The prevalence of bacteraemia was 46.5%. Gram positive bacteria, specifically *Staphylococcus* species, were the most common pathogens isolated from the bacteremic group. 42 patients survived to hospital admission. The multivariate analysis revealed that there was no association between bacteraemia and hospital mortality in OHCA patients (OR = 1.3, 95% CI= 0.2–9.2) with a *p*-value of .8. There was no association between antibiotic administration during resuscitation and hospital mortality (OR = 0.6, 95% CI= 0.1 − 3.8) with a *p*-value of .6.

**Conclusion:**

In our study, the prevalence of bacteraemia among OHCA patients presenting to the ED was found to be 46.5%. Bacteremic and non-bacteremic OHCA patients had similar initial baseline characteristics and laboratory parameters except for higher serum creatinine and BUN in the bacteremic group. In OHCA patients who survived their ED stay there was no association between hospital mortality and bacteraemia or antibiotic administration during resuscitation. There is a need for randomised controlled trials with a strong patient oriented primary outcome to better understand the association between in-hospital mortality and bacteraemia or antibiotic administration in OHCA patients.

**KEY MESSAGES**
- We aimed to determine the prevalence of bacteraemia in OHCA patients presenting to the Emergency Department. In our study, we found that 46.5% of patients presenting to our ED with OHCA were bacteremic.
- Bacteremic and non-bacteremic OHCA patients had similar initial baseline characteristics and laboratory parameters except for higher serum creatinine and BUN in the bacteremic group.
- We found no association between bacteraemia and hospital mortality. There was no association between antibiotic administration during resuscitation and hospital mortality.
- There is a need for randomised controlled trials with a strong patient oriented primary outcome to better understand the association between in-hospital mortality and bacteraemia or antibiotic administration in OHCA patients.

## Introduction

Despite advances in cardiopulmonary resuscitation (CPR), out-of-hospital cardiac arrest (OHCA) remains one of the most common causes of death globally [1]. A systematic review of the global survivorship of all-cause OHCA reported a weighted Emergency Department (ED) survival rate of 22%, and a weighted in-hospital survival rate of 8.8% [2].

The majority of OHCA cases have a cardiac origin but non-cardiac causes of OHCA do exist [3]. Numerous studies have shown an association between sepsis, bacteraemia and cardiac arrest [4,5]. This association is more prominent in OHCA compared with in-hospital cardiac arrest (IHCA) [5]. A previous study of patients with OHCA presenting to the ED found that 38% were bacteremic; notably, the ED mortality rate was higher in patients with bacteraemia than in patients without bacteremia [6]. The association between bacteraemia and cardiac arrest is topic of active research. Some studies suggest that bacteraemia may be a sequela of the invasive resuscitation measures and potential bacterial translocation from the GI tract secondary to ischemia [6,7]. Other studies suggest that bacteraemia can be a non-cardiac origin of cardiac arrest [3–5].

Despite these findings, there is a scarcity of evidence concerning the prevalence of bacteraemia in cardiac arrest patients presenting to the ED. Examining the association between bacteraemia and OHCA can have significant therapeutic and outcome implications by improving the chain of survival in the post resuscitation period. The aims of our study were to: determine the prevalence of bacteraemia in OHCA patients presenting to the ED and study the association between bacteraemia and in-hospital mortality in OHCA patients. In addition, the association between antibiotic use during resuscitation and in-hospital mortality was examined.

## Methods

### Setting

In Lebanon (setting of our study), several aspects of the American Heart Association (AHA) chain of survival are absent [8]. Prehospital emergency resources are scarce. In the prehospital setting, the management of OHCA patients by emergency medical services (EMS) is based on quick transport to the hospital and minimal medical care (first aid intervention) [8]. All the OHCA patients receive resuscitation in the Emergency Department where advanced cardiac life support (ACLS) care begins [9] (Table 1).

**Table 1.** Patient characteristics and prehospital variables.

*Continuous variables are reported as Mean ± SD, categorical variables as *N*(%)			
Patient characteristics	Bacteremic (*n* = 93)	Non-bacteremic (*n* = 107)	*p*-Value
Age	71.3 ± 16.9	70.1 ± 17.3	.6
Gender: Female	26 (28.0)	34 (31.8)	.6
Male	67 (72.0)	73 (68.2)	
Hypertension	59 (63.4)	64 (59.8)	.6
Coronary Artery Disease	40 (43.0)	41 (38.3)	.5
Atrial Fibrillation	6 (6.5)	7 (6.5)	.9
Diabetes Mellitus	32 (34.4)	34 (31.8)	.7
Dyslipidemia	18 (19.4)	24 (22.4)	.6
Chronic Heart Failure	19 (20.4)	18 (16.8)	.5
Active Malignancy	12 (12.9)	17 (15.9)	.6
Chronic Kidney Disease	10 (10.8)	12 (11.2)	.9
Chronic Obstructive Pulmonary Disease /Emphysema	5 (5.4)	13 (12.1)	.9
Cerebrovascular Accident	8 (8.6)	6 (5.6)	.4
Interval Time (Arrest to ED presentation) (min)	30.6 ± 19.4	26.4 ± 19.9	.2
Location of the arrest			.5
Home/Residence	74 (79.6)	74 (69.2)	
Public building/ Street/Highway	16 (17.2)	24 (22.4)	
Others*	3 (3.3)	9 (8.4)	
Arrest witnessed	73 (80.2)	89 (84.8)	.4
Bystander CPR initiated	88 (94.6)	93 (86.9)	.1
AED usage			
AED used	41 (50.6)	39 (48.8)	.8
AED not used	40 (49.4)	41 (51.2)	
First rhythm(prehospital)			
Shockable Rhythm	11 (34.4)	6 (30.0)	.9
Non-shockable Rhythm	19 (59.4)	12 (60.0)	
Missing Rhythm	2 (6.3)	2 (10.0)	
ROSC achieved (prehospital)	5 (5.4)	2 (1.9)	.3
Acute Coronary Syndrome symptoms			
Presyncope	10 (10.8)	13 (12.1)	.8
Gastrointestinal symptoms	15 (16.1)	20 (18.7)	.6
Diaphoresis	5 (5.4)	19 (17.8)	.007
Shortness of breath	26 (28.0)	43 (40.2)	.07
Pain or discomfort in the chest	17 (18.3)	30 (28.0)	.1
Pain or discomfort elsewhere	6 (6.5)	11 (10.3)	.3
Other	1 (1.1)	7 (6.5)	.07
Infection variables			
Current antibiotic use	8 (10.3)	12 (13.0)	.6
Pulmonary infection	19 (20.4)	26 (24.3)	.5
Fever	9 (9.7)	14 (13.1)	.5
Urinary tract infection	4 (4.3)	6 (5.6)	.8
Gastrointestinal infection	7 (7.5)	10 (9.3)	.7
Skin infection	3 (3.2)	1 (0.9)	.3

Table 1 shows the patient characteristics and prehospital variables of both bacteremic and non-bacteremic out of hospital cardiac arrest patients.

ED: emergency department; AED: automated external defibrillator; CPR: Cardiopulmonary Resuscitation; ROSC: Return of Spontaneous Circulation.

The study was performed at an academic tertiary care centre in Lebanon with greater than 50,000 annual ED patient visits [10].

### Inclusion and exclusion criteria

This was a single centre, observational study. All adult patients (≥18 years) who presented to the ED of our tertiary care medical centre with OHCA between October 2015 and August 2019 and underwent resuscitation in the ED were included. Research assistants present in the hospital were informed about eligible patients by the treating team. Patients were recruited 24 h a day, 7 days a week. Patients excluded from our study included: patients who refused consent, patients under the care of any member of the research team (to avoid observer bias), pregnant women, OHCA due to trauma or electrocution, patients with resuscitative efforts that were discontinued due to perceived futility of efforts (rigor mortis, lividity), patients with less than two sets of blood cultures and patients below the age of 18 years. All patients presenting to the ED with OHCA received Cardiopulmonary Resuscitation (CPR). OHCA was diagnosed based on the American Heart Association (AHA) and the International Consensus Conference on Cardiopulmonary Resuscitation (CPR) guidelines [11]. All resuscitations performed by the ED team were in accordance with the advanced cardiac life support (ACLS) guidelines. All patients received the same standard of care provided for all OHCA victims presenting to the ED without involvement of any member of the research team. Patients who were under the care of any member of the research team were not approached to be included in the study to avoid observer bias. The research assistants consented the families of all included patients. The consent was informed, voluntary and written. This study was approved by our hospital Institutional Review Board (IRB# ER.GA.04)

### Blood sample collection and patient classification

The study involved drawing blood for analysis and culture (aerobic and anaerobic) on OHCA patients undergoing resuscitation in the ED. Blood withdrawal was performed during active resuscitation by a registered nurse or phlebotomist who was not part of the resuscitation team. Blood cultures were collected during resuscitation from two different sites to decrease the chance of skin flora contamination (aerobic and anaerobic blood cultures from two different sites). In an effort to limit contamination, the individual performing the blood culture collection washed his/her hands and wore clean non-sterile gloves, the caps of the anaerobic and aerobic bottles were removed and the septum disinfected with 70% alcohol swabs, the skin over the venipuncture site was then cleaned with chlorhexidine and allowed to dry. While waiting for the skin to dry the individual collecting the blood culture discarded the non-sterile gloves, washed his/her hands again and wore sterile gloves before finally blood was drawn using disposable sterile syringes and needles. A standard definition of positive blood cultures was used to identify patients with bacteraemia^1^. Patients were then divided into two groups based on culture results: bacteremic OHCA patients and non-bacteremic OHCA patients.

### Variables and outcomes

The following information from OHCA patients were collected from the electronic medical records: vital signs upon presentation to ED, medical history, laboratory parameters, two blood cultures, urine analysis and urine cultures, medications used during resuscitation (including use of vasopressors, inotropes, antibiotics and steroids), patient disposition and presence or absence of bacteraemia and mortality at 72 h and 28 days. The primary outcome was the prevalence of bacteraemia. The secondary outcome was in hospital mortality.

### Statistical analysis

Student *t*-test was used to compare the differences in continuous variables, and the *χ*^2^ test was used to compare the differences in categorical variables between both groups (Bacteremic vs non-bacteremic). In the bivariate analysis, the statistical association between the independent variables and the dependent variable mortality was assessed. In the multivariate analysis, logistic regression was used to assess the magnitude of association between bacteraemia or antibiotic administration and hospital mortality. The multivariate logistic regression only included the 42 OHCA patients who survived their ED stay. Missing data were not handled through multiple imputations because the percent of missing values in all variables was found to be less than 5%. All the tests were interpreted at a predetermined significance level (*α* = 0.05). Statistical analyses were performed using SPSS Statistics for Windows V.21.0. (Armonk, New York, USA: IBM Corp).

## Results

**Figure 1.**
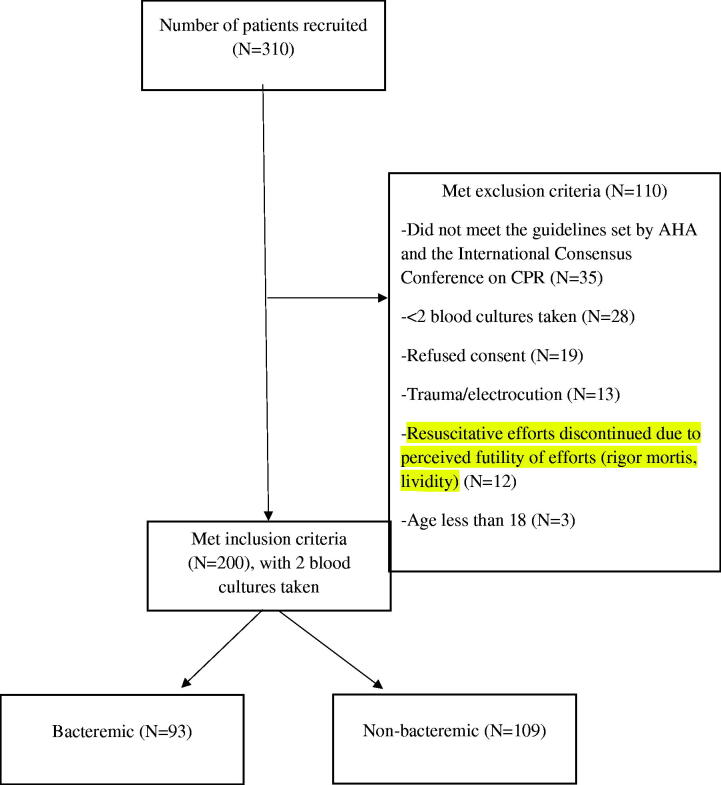
Flowchart.

We recruited a total of 310 OHCA patients from October 2015 to August 2019 (Figure 1). 110 patients met the exclusion criteria, and 200 patients remained and were followed for a period of one month. Out of the 200, 93 were considered bacteremic. The prevalence of bacteraemia was 46.5% over the 4-year study period.

There were no significant differences in gender, age or medical history between bacteremic and non-bacteremic OHCA patients. The two groups had similar initial laboratory parameters except for higher serum creatinine and BUN in the bacteremic group (Table 2).

**Table 2.** Initial rhythm and lab parameters upon presentation to the ED.

*Continuous variables are reported as Mean ± SD, categorical variables as *N*(%)			
Lab parameters upon presentation to the ED	Bacteremic (*n* = 93)	Non-bacteremic (*n* = 107)	*p*-Value
Initial Rhythm in the ED			
Shockable Rhythm	12 (12.9)	10 (9.3)	.3
Non-shockable Rhythm	77 (82.8)	87 (81.3)	
Missing Rhythm	4 (4.3)	10 (9.3)	
Labs			
WBC/cu.mm	19398 ± 43771	15618 ± 16723	.4
BUN (mg/dL)	42 ± 34	30 ± 23	.003
Creatinine (mg/dL)	2.2 ± 1.8	1.5 ± 0.9	.002
Sodium (mmol/L)	140.8 ± 11	141.3 ± 10.7	.8
Potassium (mmol/L)	6.2 ± 1.5	5.9 ± 1.6	.1
Chloride (mmol/L)	97.8 ± 9.0	97.3 ± 8.6	.7
Bicarbonate (mmol/L)	17.6 ± 7.9	18.1 ± 7.2	.7
Magnesium (mg/dL)	3.1 ± 1.7	2.7 ± 0.6	.02
Phosphate (mg/dL)	8.5 ± 3.5	8.1 ± 2.5	.4
Lactic Acid (mmol/L)	14.3 ± 5.6	14.3 ± 5.5	.9
Troponin T (ng/ml)	0.3 ± 0.6	0.2 ± 0.2	.09
pH Arterial	7.1 ± 0.2	7.0 ± 0.2	.1
PaCO2 (mmHg)	66.3 ± 36.9	78.5 ± 59.2	.2

Table 2 shows the initial rhythm and lab parameters upon presentation to the emergency department of both bacteremic and non-bacteremic out of hospital cardiac arrest patients.

ED: emergency department; WBC: White Blood Cells.

There was no significant difference in CPR duration in the ED between bacteremic and non-bacteremic OHCA patients. Therapeutic interventions, including intravenous (IV) fluid volumes and vasopressor use, were similar between groups (Table 3).

**Table 3.** Therapeutic parameters and outcomes of patients.

*Continuous variables are reported as Mean ± SD, categorical variables as *N*(%)			
Therapeutic parameters and outcomes of patients	Bacteremic (*n* = 93)	Non-bacteremic (*n* = 107)	*p*-Value
Total epinephrine during resuscitation (mg)	7 ± 6	7 ± 6	.3
Number of shocks	6 ± 8	4 ± 4	.2
CPR time	23.3 ± 10.7	22.9 ± 10.1	.8
Time to first antibiotic (min)	81.0 ± 73.8	93.1 ± 61.3	.8
Time to vasopressor/inotrope initiation (min)	40.9 ± 24.5	33.9 ± 26.2	.3
Vasopressor/inotrope duration (h)	37.1 ± 57.3	41.0 ± 55.9	.8
IV fluid requirement first 6 h (L)	1.5 ± 1.9	1.3 ± 0.8	.08
IV fluid requirement first 24 h (L)	1.7 ± 1.3	1.65 ± 1.29	.8
ROSC in ED	30 (34.1)	50 (44.6)	.1
Temperature after ROSC achieved (°C)	36.7 ± 1.1	36.0 ± 1.0	.5
Systolic blood pressure after ROSC achieved (mmHg)	106.3 ± 35.1	113.3 ± 37.7	.4
Diastolic blood pressure after ROSC achieved (mmHg)	58.7 ± 28.3	67.2 ± 24.4	.2
Heart Rate after ROSC achieved (bpm)	110.3 ± 33.9	102.7 ± 29.5	.3
Respiratory rate after ROSC achieved (/min)	24.1 ± 7.6	20.6 ± 6.4	.06
Oxygen saturation after ROSC achieved (%)	92.2 ± 10.1	94.7 ± 8.1	.3
Time to ROSC (min)	18.8 ± 10.1	15.6 ± 9.7	.2
Glasgow Coma Scale (GCS)			
Mild (14)	0 (0)	2 (1.9)	.5
Moderate (8–13)	0 (0)	1 (0.9)	
Severe (<8)	93 (100)	104 (97.2)	
Cerebral Performance Category (CPC)			
Good (1,2)	0 (0)	2 (1.9)	.5
Bad (3,4,5)	93 (100)	105 (98.1)	
ED mortality	80 (86.0)	78 (72.9)	.02
28-day mortality	88 (94.6)	97 (90.7)	.4
Hospital mortality	90 (96.8)	97 (90.7)	.08
Disposition			
Discharged home or to another facility	0 (0)	3 (2.8)	.4
Transferred to another acute care facility from ED or Floor	3 (3.2)	5 (4.7)	
Morgue	90 (96.8)	102 (90.7)	
Against Medical Advice	0(0)	2 (1.9)	

Table 3 shows the therapeutic parameters and outcomes of both bacteremic and non-bacteremic out of hospital cardiac arrest patients. ED: Emergency Department; CPR: Cardiopulmonary Resuscitation; ROSC: Return of Spontaneous Circulation; IV: Intravenous.

Post-return of spontaneous circulation (ROSC) variables did not differ between both groups. ED mortality was higher in the bacteremic patients compared to the non bacteremic patients (86.0% versus 72.9% respectively) with a *p*-value of .02. However, there was no significant difference in in-hospital or 28-day mortality between the two groups (Table 3). The most common pathogen isolated from culture was *Staphylococcus* (47% of positive cultures), followed by *Enterococcus* (10.7%) and *Escherichia coli* (10.7%) (Table 4).

**Table 4.** Organisms found in the blood and in the urine.

Bacteraemia	Count (*N* = 93)	Column %
*Other Staphylococcus species*	28	30.1
*Staphylococcus epidermidis*	16	17.2
*Escherichia coli*	10	10.7
*Enterococcus species*	10	10.7
*Other Streptococcus species*	8	8.6
*Streptococcus viridans*	5	5.3
*Staphylococcus aureus*	3	3.2
*Streptococcus aglactiae*	3	3.2
*Pseudomonas aeruginosa*	2	2.2
*Streptococcus pneumoniae*	1	1.1
*Acinetobacter baumannii*	1	1.1
*Hemophilus species*	1	1.1
*Proteus mirabilis*	1	1.1
*Clostridium species*	1	1.1
*Corynebacterium jeikeium*	1	1.1
*Klebsiella pneumoniae*	1	1.1
*Morganella morganii*	1	1.1
Urinary Tract Infection*	Count (*N* = 20)	Column %
*Escherichia coli*	14	70
*Pseudomonas aeruginosa*	2	10
*Enterococcus species*	2	10
*Klebsiella pneumoniae*	2	10

Table 4 shows the organisms that were isolated in the blood and urine cultures of the bacteremic out of hospital cardiac arrest patients.

Results of the multivariate analysis revealed that there was no association between bacteraemia and hospital mortality in OHCA patients (OR = 1.3, 95% CI= 0.2–9.2) with a *p*-value of .8. There was no association between antibiotic administration during resuscitation and hospital mortality (OR = 0.6, 95% CI= 0.1 − 3.8) with a *p*-value of .6 (Table 5).

**Table 5.** Logistic regression for Hospital Mortality.

Hospital mortality (reference: No)		
Variables	OR ( 95 % CI )	*p*-Value
Bacteremic	1.3 (0.2 − 8.2)	.8
Antibiotic given during resuscitation	0.6 (0.1 − 3.8)	.6
Lactate	1.2 (1.0 − 1.5)	.1
Variables included in the model were: Bacteremic (reference: no); Age (reference: below 73), gender (reference: female), magnesium, Diabetes (reference: no), Dyslipidemia (reference: no), Cardiac complications (reference:no), antibiotic given during resuscitation (reference:no).		

Table 5 shows the multivariate logistic regression showing no association between bacteraemia, antibiotic use during resuscitation, lactate level and hospital mortality in out of hospital cardiac arrest patients who survived their emergency department stay.

## Discussion

In this single-centre study of patients presenting to the ED following OHCA, we found a 46.5% prevalence of bacteraemia. Gram positive bacteria, specifically *Staphylococcus* species, were the most common pathogens isolated from the bacteremic group. After adjusting for confounders, the multivariate analysis revealed that there was no association between bacteraemia and hospital mortality in OHCA patients. There was no association between antibiotic administration during resuscitation and hospital mortality.

### The impact of bacteraemia

In our study, the prevalence of bacteraemia in OHCA patients was 8.5% higher compared to Coba et al. (38% of 173 OHCA patients) [6]. The association between bacteraemia and OHCA mortality has the potential to carry major therapeutic implications. It has been shown previously that undetected bacteraemia with inadequate treatment is strongly associated with increased mortality and long-term morbidity in septic patients [12]. As well, the administration of early antibiotics in sepsis/septic shock has been definitively shown to be beneficial [13]. Therefore, it is possible that early recognition of sepsis as the cause of OHCA and subsequent antibiotic administration might improve the survival rate in those patients who survive initial emergency department resuscitation.

Several studies examined infections in the post-resuscitation period. Gajic et al. reported that 46% of the OHCA patients admitted to the Intensive care unit (ICU) post CPR developed a new infection [14]. Patients who developed an infection had increased length of mechanical ventilation, ICU length of stay and hospital mortality. In a study by Mongardon et al. 67% of OHCA patients developed infectious complications post resuscitation [15]. ICU mortality was similar between both infected and non-infected patients [15]. These two studies focussed on infectious complications that occurred after resuscitation was successful. They did not address the possible presence of infection prior to the cardiac arrest and that an infection may have potentially contributed to the cardiac arrest itself.

Gaussorgues et al. found that most of the pathogens isolated 12 h post resuscitation were *Streptococcus* (38.4%) and Gram negatives (46.1%). These findings can be explained by the low cardiac output secondary to the cardiac arrest which led to mesenteric ischaemia causing gut flora to move into the blood leading to sepsis [7]. Contrary to Gaussorgues et al. most of the blood cultures we obtained grew *Staphylococcus* (47%). Coba et al. had similar results with their most reported pathogens being *Staphylococcus* (37%) and *Streptococcus* species (27.5%) [6]. This discrepancy can be due to the time the blood cultures were taken; immediately upon arrival to the ED, the closest time possible to the time of arrest [6].

### Factors associated with mortality in OHCA patients

#### Bacteraemia in OHCA patients

There is controversy on the association between bacteraemia and cardiac arrest. Some studies suggest that bacteraemia is a consequence of the invasive resuscitation measures and potential gastrointestinal bacterial translocation [6,7]. Other studies state that bacteraemia is one of the non-cardiac origins of cardiac arrest [3–5]

Several mechanisms can explain how bacteraemia can be a non-cardiac origin of OHCA. One possible mechanism involves sepsis-induced myocardial injury and hypoxia. Sepsis is a complex process that overwhelms the immune system and triggers an augmented immune response even at sites distant from the original inflammation site. The progression to septic shock is correlated with onset of new fatal arrhythmias like ventricular and atrial fibrillations [16,17]. Sepsis-induced tissue hypoxia combined with the exposure to vasoconstrictors contribute to a decreased myocardial blood supply and gross ischaemia [18]. A prospective post-mortem examination by Schmittinger revealed stress-induced and ischaemic histopathologic changes in the myocardial and coronary arterial tissues of septic shock patients [19].

The second mechanism involves sepsis-induced direct myocardial inflammation and depression. Bacterial endotoxins, as well as pro-inflammatory cytokines and reactive oxygen species, can lead to a decrease in myocardial contractility and a worse cardiac function [20]. Moreover, Cuence et al. described the infiltration of the heart by monocytes and the expression of new extracellular matrix enzymes by the cardiomyocytes in early sepsis, leading to cardiac dysfunction [21].

#### Antibiotic administration in OHCA patients

We found that 42 patients of the 200 survived in the ED, 88% of which received antibiotics. Coba et al. reported that 69% of OHCA ED survivors received broad-spectrum antibiotics in the ED [10]. Davies et al. showed an early prophylactic treatment of OHCA patients with empiric antibiotics increased survival rate [22], and Hellenkamp et al. demonstrated a longer length of hospital and ICU stay in OHCA patients who received a delayed antibiotic treatment [23]. Gagnon et al. found that prophylactic antibiotic administration in cardiac arrest survivors was associated with less pneumonia and sepsis in the post-resuscitation period (12.6% vs 54.9% and 1.2% vs 5.7% respectively with a *p* < .001) but both groups had similar functional outcome [24]. On the other hand, Couper et al. showed that prophylactic antibiotic use in cardiac arrest patients was not associated with improved survival, better neurological outcome or decreased hospital length of stay which is in line with our results [25]. Further studies should be done to address the question of antibiotic administration.

### Limitations

This was a single centre study which could compromise the generalisability of our results to the entire population. Some of the OHCA patients were taking antibiotics prior to presentation which could have suppressed bacterial growth in culture and lead to underestimation of the prevalence of bacteraemia. Information regarding the antibiotic given during resuscitation was missing. Time to ED arrival after cardiac arrest occurrence was not included in our study which could have affected mortality. In addition, a major confounder of the study was the lack of pre-hospital resuscitation (ACLS). Pre-hospital resuscitation (ACLS) plays a crucial role in achieving ROSC in cardiac arrest patients. The delay in resuscitation due to the lack of pre-hospital ACLS could have reduced the number of patients who survived their ED stay and resulted in a relatively low number of patients included in our multivariate logistic regression (the results obtained in the multivariate analysis were not statistically significant). Although we defined bacteraemia with a common skin-flora organism in an OHCA patient as two blood culture bottles growing a skin flora pathogen from two different sites, the unknown clinical significance of positive cultures with common skin-flora organisms remains a limitation. Repeating blood cultures after the resuscitation to verify the persistence of bacteraemia was not feasible in this study and should be done in future studies. Finally, the treating physicians could have adjusted antibiotics based on the results of the blood cultures and this could have affected the mortality of the bacteremic patients.

## Conclusion

In our study, the prevalence of bacteraemia among OHCA patients presenting to the ED was found to be 46.5%. Bacteremic and non-bacteremic OHCA patients had similar initial baseline characteristics and laboratory parameters except for higher serum creatinine and BUN in the bacteremic group. In OHCA patients who survived their ED stay there was no association between hospital mortality and bacteraemia or antibiotic administration during resuscitation. There is a need for randomised controlled trials with a strong patient oriented primary outcome to better understand the association between in-hospital mortality and bacteraemia or antibiotic administration in OHCA patients. In the future we would like to consider collecting blood cultures in OHCA patients, serially at two different times (during resuscitation and after resuscitation). This can help confirm the persistence of bacteraemia with a true pathogen and compare the microorganisms identified by blood culture during and post resuscitation to better understand if bacteraemia can be considered as an origin of cardiac arrest or a consequence of cardiac arrest.

## Data Availability

All the data was presented in the form of Tables. Patient information was collected from the electronic medical files of our institution. The data that support the findings of this study are available from the corresponding author [MES], upon reasonable request.

## Note

1 An OHCA patient was identified as having bacteremia if at least one of the blood culture bottles grew a non-skin flora pathogen or if two blood culture bottles grew a skin flora pathogen from the two different sites [6].

## Study registration

This study was approved by our hospital Institutional Review Board (IRB# ER.GA.04). This study was registered on the Clinical Research Portal of the American University of Beirut. This can be accessed with the following link: https://eur03.safelinks.protection.outlook.com/?url=https%3A%2F%2Fclinicalresearch.aub.edu.lb%2FClinicalTrials%2FDetails%2F119&data=04%7C01%7Cmelsayed%40aub.edu.lb%7Cbef6325d48f74496f44e08d93714bbfe%7Cc7ba5b1a41b643e9a1206ff654ada137%7C1%7C0%7C637601384452130767%7CUnknown%7CTWFpbGZsb3d8eyJWIjoiMC4wLjAwMDAiLCJQIjoiV2luMzIiLCJBTiI6Ik1haWwiLCJXVCI6Mn0%3D%7C1000&sdata=aJaFLyUKUXmzIVK2i7WtYdq4D67iSe66jJws%2FcRe074%3D&reserved=0

## Abbreviations

**Abbreviations**
OHCA: Out-of-hospital cardiac arrest
ED: Emergency Department
CPR: Cardiopulmonary Resuscitation
IHCA: In-Hospital Cardiac Arrest
AHA: American Heart Association
EMS: Emergency Medical Services
ACLS: Advanced Cardiac Life Support
AED: Automated External Defibrillator
ROSC: Return of Spontaneous Circulation
IV: Intravenous
ICU: Intensive Care Unit
